# Design and characterization of diclofenac diethylamine transdermal patch using silicone and acrylic adhesives combination

**DOI:** 10.1186/2008-2231-21-6

**Published:** 2013-01-07

**Authors:** Dandigi M Panchaxari, Sowjanya Pampana, Tapas Pal, Bhavana Devabhaktuni, Anil Kumar Aravapalli

**Affiliations:** 1 Department of Pharmaceutics, KLEU’s college of Pharmacy, Nehru Nagar, Belgaum, Karnataka, 590010, India; 2 Research Scientist, R&D divison, Sparsha Pharma International Pvt. Ltd., Hyderabad, India

**Keywords:** Transdermal drug delivery system, Silicone adhesive, Acrylic adhesive, Permeation study, Dissolution, Skin irritancy and anti-inflammatory

## Abstract

**Background and purpose of the study:**

The objective of the study was to develop and characterize Diclofenac Diethylamine (DDEA) transdermal patch using Silicone and acrylic adhesives combination.

**Methods:**

Modified solvent evaporation method was employed for casting of film over Fluoropolymer coated polyester release liner. Initial studies included solubilization of drug in the polymers using solubilizers. The formulations with combination of adhesives were attempted to combine the desirable features of both the adhesives. The effect of the permeation enhancers on the drug permeation were studied using pig ear skin. All the optimized patches were subjected to adhesion, dissolution and stability studies. A 7-day skin irritancy test on albino rabbits and an in vivo anti-inflammatory study on wistar rats by carrageenan induced paw edema method were also performed.

**Results:**

The results indicated the high percent drug permeation (% CDP-23.582) and low solubility nature (1%) of Silicone adhesive and high solubility (20%) and low% CDP (10.72%) of acrylic adhesive. The combination of adhesives showed desirable characteristics for DDEA permeation with adequate % CDP and sufficient solubility. Release profiles were found to be dependent on proportion of polymer and type of permeation enhancer. The anti-inflammatory study revealed the sustaining effect and high percentage inhibition of edema of C4/OLA (99.68%). The acute skin irritancy studies advocated the non-irritant nature of the adhesives used.

**Conclusion:**

It was concluded that an ideal of combination of adhesives would serve as the best choice, for fabrication of DDEA patches, for sustained effect of DDEA with better enhancement in permeation characteristics and robustness.

## Introduction

Drugs can be delivered across the skin to have an effect on the tissues adjacent to the site of application (topical delivery) or to have an effect after distribution through the circulatory system (systemic delivery). While there are many advantages for delivering drugs through the skin the barrier properties of the skin provide a significant challenge. By understanding the mechanisms by which compounds cross the skin it will be possible to devise means for improving drug delivery [1]. In the last decades, transdermal dosage forms have been introduced for providing a controlled delivery via the skin into the circulation system.

A transdermal patch or skin patch is a medicated adhesive patch that is placed on the skin to deliver a specific dose of medication through the skin and into the blood stream. Drug-in-adhesive-type patches have been gaining increasing popularity as effective transdermal delivery systems during the last two decades [2] due to various advantages over other systems namely, they are easy to construct, less chances of dose dumping and patches with less thicknesses can be prepared.

Diclofenac is a well-established non-steroidal anti-inflammatory agent, widely used in musculoskeletal disorders, arthritis, toothache, dysmenorrhea, etc., for symptomatic relief of pain and inflammation [3]. Diethylammonium salt of Diclofenac (Diclofenac Diethylamine) is reportedly used for topical applications. Diclofenac Diethylamine (DDEA) gel (1.16%; Voltaren® Emulgel®, Novartis, Nyon, Switzerland) has been used extensively in Europe since 1985 to relieve the symptoms of OA of the knee, as well as other painful, inflammatory tendon, ligament, muscle, and joint conditions [4]. However, all NSAIDs include a boxed warning highlighting the potential for increased risk of cardiovascular events as well as serious potential life-threatening gastrointestinal bleeding. The drug undergoes substantial hepatic first-pass metabolism and thus only about 50% of the administered dose reaches systemic circulation [3,5]. This originates the need of an alternative route of administration, which can bypass the hepatic first-pass metabolism. Transdermal route is an alternative choice of route of administration for such drugs. The drug, Diclofenac Diethylamine also possesses the ideal characteristics, such as poor bioavailability (40 to 60%), short biological half-life (2 to 3 h), smaller dose (25 to 50 mg), etc., to be formulated into a transdermal patch. Transdermal patches offer added advantages, such as maintenance of constant and prolonged drug level, reduced frequency of dosing, minimization of inter and intra patient variability, self-administration and easy termination of medication, leading to patient compliance [6].

It has been postulated that Diclofenac transdermal exerts its pharmacological effects through localized accumulation at the site of application rather than from the systemic absorption. The bioavailability of Diclofenac transdermal is approximately 1% that of oral Diclofenac, with an elimination half-life of 12 h compared with 1.2 to 2 h with oral Diclofenac [7].

The present study aimed at developing TDDS drug-in-adhesive patches of DDEA using Silicone adhesives, Acrylic adhesives and blend of Silicone and Acrylic adhesives.

## Material and methods

### Materials

The Silicone polymers (S_1_ to S_6_) were purchased from Dow Corning Corporation, (midland, MIA, USA), Acrylic polymer (A) was purchased from National Starch and Chemical company (Bridge Water, NJ, USA). Fluoropolymer coated polyester release liner and Polyester Backing laminate was purchased from 3 M Scotchpak (st. paul, USA). The drug Diclofenac Diethylamine B.P (DDEA) was obtained from Sparsha Pharma International Pvt Ltd (Hyd, India). Methanol and Acetonnitrile were of HPLC grade and purchased from Sigma-Aldrich corporation, India. All other reagents used were of highest reagent grade available.

### Preparation of patches containing silicone adhesives

#### Preparation of placebo silicone patches

Transdermal patches were prepared by modified solvent evaporation method. It is similar to conventional method except, that the drug-polymeric solution was spread over the release liner with the help of manual coater over release liner. Transdermal patches using different silicone polymers (Table 1) without drug were prepared. For preparing transdermal patches, an adequate amount of polymeric solution was taken and then spread over the release liner with the help of a manual coater. The polymeric solution coated liner was dried at 80°C in an oven for 10 min. The patches were then finally laminated with polyester backing membrane. The obtained sheets were punched using suitable dyes (3, 10 and 50 cm^2^) to get patches of appropriate sizes, packed in aluminum foil and stored in a desiccator for further studies. Patches were prepared using different grades of silicone adhesives.

**Table 1:** Details of silicone and acrylic polymers used in the study

**Code**	**Functional group**	**Solvent**	**Solid content (%)**	**Viscosity (Mpa.s)**
*Silicone Polymer*				
S1	Amine compatible	Ethyl acetate	60	350
S2	Amine compatible	Ethyl acetate	60	800
S3	Amine compatible	Ethyl acetate	60	1200
S4	-	Ethyl acetate	60	650
S5	-	Ethyl acetate	65	2500
S6	-	Ethyl acetate	60	2600
*Acrylic polymer*				
A, Polyacrylate	COOH	Ethylacetae and Hexane	43.2	7000-19000

### Physical evaluation of placebo patches

#### The tack of the patches – ball tack test

Tack is the ability of a pressure-sensitive adhesive to bond under conditions of light contact pressure and a short contact time. The tack of the skin contact adhesive was measured by the rolling ball tack test using primary adhesive tester (Labthink Instruments Co. Ltd., China). The patch with a size of 50 cm^2^ was fixed on a plate. Different diameter steel balls were released from the top of the inclined plate (angle 45°C). The number of the largest ball (0 – 9) which did not roll down was reported as the tack value [8].

#### Peel strength of patches

Peel strength measures the force required to peel away a pressure-sensitive adhesive once it has been attached to a surface. The test was performed with a Digital Peel tester with a load capacity up to 5 kg (Make: International Equipments, Model: CO). A piece of the patch which has a width of 10 mm and length of 25 mm was prepared, applied quickly to the end of the stainless steel plate and left the apparatus for 10 min.

The cello tape was affixed on the product. The free end of this tape was bending back 180° and it was attached firmly to the upper part of a peel testing machine with a clamp. The instrument was started with a speed of 300 mm/min and the values were recorded. Five patches form each batch were used measuring strength and their values were averaged [8].

### Preparation of drug loaded silicone patches

#### Solubility of drug in adhesives

The solubility of drug in adhesive was tested in silicone adhesives (S_3_ and S_6_). Different concentrations of drug (5% w/w, 3% w/w, 2% w/w and 1% w/w of final patch formulation) were added to the adhesives under constant stirring with the help of magnetic stirrer. The stirring was continued for a period of 4 h in order to ensure complete mixing. The solution was kept aside overnight for visual observation. The solutions that showed turbidity were discarded and solutions that remained clear were coated over release liner and finally laminated with backing layer as described in the section 2.2.1.

#### Solubility enhancement techniques

Various solubility enhancements used to increase the solubility of Diclofenac Diethylamine in silicone adhesives include

Addition of solubilizers

Polyethylene glycol – 400 (PEG 400) and Propylene glycol (PG) in different concentrations (% w/w of final patch formulation) were used [9]. The adhesive polymeric solution, drug and solubilizer in required quantities were weighed and mixed with the aid of magnetic stirrer for a period of about 4–5 h. The solutions were monitored visually for appearance of turbidity/sedimentation. The formulations that showed clear solution after 24 h were coated over release liner and laminated with polyester backing laminate. The patches were packed in aluminum foil, kept aside for 10 days for appearence of crystals visually and microscopically. The patches which did not show crystals after 10 days were selected for further study.

Addition of oils

Four oils were slected based on preliminary study to improve the solubility of Diclofenac Diethylamine [10]. The oils, Oleic acid (OLA), Iso stearic acid (ISA), Pharamasolve (PS) and Iso propyl myristate (IPM) in different ratios of drug: solubilizer were mixed with polymeric solution and then monitored visually for turbidity. The clear solutions were used for preparation of patches which were kept aside for 10 days for appearance of crystals.

### Ex vivo skin permeation studies

#### Preparation of skin barrier

Fresh full-thickness (75–80 mm) pig ear skin was used for the study. The experiment was carried out according to the guidelines of the Committee for the Purpose of Control and Supervision of Experiments on Animals and approved by Animal Ethical Committee of Department of Genetics, Osmania University, Hyderabad, India (approval no.380/01/a/CPCSEA). Fresh pig ears were obtained from a local abattoir; to ensure integrity of the skin barrier, ears were removed post-sacrifice. The skin was dermatomed (Zimmer electric Dermatome Handset) to remove dermis [11,12]. The isolated epidermis (100 μm) was rapidly rinsed with hexane to remove surface lipids and then rinsed with water and used immediately.

The *ex vivo* skin permeation from the prepared drug polymeric patches across the porcine ear skin barrier was studied using Franz diffusion cell (Orchid Scientifics & Innovative India Pvt Ltd.), [13,14]. Twenty - five milliliters of phosphate buffer of pH 7.4 was used as an elution medium. The diameter of the donor compartment cell provided an effective constant area of 3.4 cm^2^. The dermatomed pig ear skin was mounted between the two compartments of Franz diffusion cell with stratum corneum facing towards the donor compartment. A 3 cm^2^ patch was used for the study. The release liner was removed. The patches to be studied were placed in between the donor and the receptor compartment in such a way that the drug releasing surface faced toward the receptor compartment. After securely clamping the donor and receptor compartments together, the elution medium was magnetically stirred for uniform drug distribution at a speed of 60 rpm. The temperature of the whole assembly was maintained at 32 ± 0.5°C by thermostatic arrangements. An aliquot of 0.5 mL was withdrawn at preset time intervals for a period of 24 h and an equivalent volume of fresh buffer was replaced. The samples removed were analysed by HPLC described below.

### Preparation of patches containing acrylic adhesive

The formulations containing different concentrations of drug with acrylic adhesive were prepared by the method described under section 2.2. The patches prepared were monitered for appearance of crystals visually for 10 days. The properties of acrylic adhesive were mentioned in the Table 1. The patches which showed stability were subjected to peel test, ball test (described under 2.2) and permeation study (described in section 2.3).

### Preparation of patches containing combination of silicone and arylic adhesives

Placebo patches containing combination of silicone and acrylate adhesives in different ratios and drug containing combinational patches were prepared by the following method:

In First step, required amount of drug (% w/w of final patch formulation) was made to dissolve completely in appropriate amount of acrylate adhesive by continuous stirring. Second step involves addition of silicone polymeric solution to clear solution formed in step 1 and then continuing mixing for a period of 12 h. The formulations that showed drug solubility after 24 h were laminated into patches. The patches which showed stability were subjected to peel test, ball test (described under 2.2) and permeation study (described in section 2.3).

### Effect of permeation enhancers on drug loaded combinational patches

The incorporation of a permeation enhancer is indispensable for achieving the desired permeation rate for almost all drugs with the limited size of the patch. The permeation enhancers Oleic acid (OLA), Iso Stearic acid (ISA) and Isopropyl Myristate (IPM) at concentraions of 5% each were chosen to study their effect on permeation of Diclofenac Diethylamine across the skin. The solubilized combinational patches (C_4_ and C_5_) along with different permeation enhancers (5% concentration) were formulated and subjected for permeation study as described under 2.3.

### Characterization of optimized patches

Various physicochemical tests employed for optimized transdermal patches were as shown

#### Thickness

Patch thickness was measured using digital micrometer screw gauge (Mitutoyo, Japan) at five different places. The average and standard deviation of five readings were calculated for each batch of the drug-loaded films.

#### Weight uniformity

Five different films from individual batches were weighed individuall, and the average weight was calculated the individual weight should not deviate significantly from the weight was calculated, the individual weight should not deviate significantly from the average weight, so the standard deviation was calculated [15].

#### Drug content

Assay of Diclofenac Diethylamine was done with the help of HPLC. All the solvents used were of HPLC grade [16].

Sample solution

For determination of drug content one patch of 50 cm^2^ was taken, dissolved in HPLC grade methanol and sonicated for 15 min. From above solution 1 mL was taken into a 50 mL volumetric flask, diluted up to the mark with methanol, filtered through Nylon membrane filters of 0.45 μ size (Pall Pharmalab Filtration Pvt. Ltd.) and injected (20 mL) into the HPLC column.

Standard solution

For preparing standard solution, 50 mg of Diclofenac Diethylamine was dissolved in 50 mL methanol (HPLC grade). From the above solution, 1 mL was taken and diluted to 50 mL with methanol which was finally filtered through a Nylon membrane filters of 0.45 μ size (Whatman GF/C) and injected (20 mL) into the HPLC column.

HPLC conditions

The HPLC system consisted of L-7110 pump (Shimadzu Corporation, Japan) with L-7420 variable-wavelength ultraviolet absorbance detector (Shimadzu Corporation, Japan) set at 274 nm. Analysis was performed on a reversed-phase column made of silica (150 mm × 4.6 mm i.d., 5 μm, Chemsil BDS C18, Beijing China), operated at 40°C. The mobile phase consisted of 45: 55 ratio of 0.5% Glacial acetic acid in water and Acetonitrile. HPLC grade water was used for the preparation of 0.5% Glacial acetic acid solution. The flow rate of mobile phase was set at 0.8 mL/min, was used. The injection volume is 20 μL.

#### In vitro release – dissolution studies

The release-rate determination is one of the most important studies to be conducted for all controlled release delivery systems. The dissolution studies of patches are very crucial, because one needs to maintain the drug concentration on the surface of stratum corneum consistently and substantially greater than the drug concentration in the body, to achieve a constant rate of drug permeation [17].

A Paddle over disc assembly (USP 23, Apparatus 5) was used for the assessment of release of DDEA. The TDDS patch was mounted on the disc and placed at the bottom of the dissolution vessel. The dissolution medium, 900 ml degassed distilled water at pH 7.0. The apparatus was equilibrated to 32 ± 0.5°C and operated at 50 rpm [18] during the entire study period (24 h). The dissolution medium was degassed by a combination of heating up to 45°C and vacuum filtration followed by vigorous stirring of media under vacuum.

#### Stability study

The optimized formulations were subjected to stability study by storing patches at 40 ± 2°C and 75% RH in stability chamber for three months. Two parameters namely, peel strength and drug content were analyzed.

#### Surface morphology

The surface morphology of formulated transdermal patches (both stable and unstable) were investigated by using Scanning electron microscope (model: SEM JSM-6610) at 15 kV under different magnifications (950x, 1000x and 1500x). In order to make the samples electrically conductive the samples were gold coated prior to the study.

#### Acute skin irritancy test

The study was conducted on the basis of the approval of institutional animal ethical committee. Albino rabbits of either sex, each weighing 1.5 to 2.0 kg, divided into two groups, were used in this study (n = 4 in each group) [14]. They were housed in cages in the animal house under controlled temperature and light conditions. They were fed a standard laboratory diet and had access to water *ad libitum*. The dorsal surface of the rabbits was cleared and the hair was removed by shaving. The skin was cleared with rectified spirit. The experimental patch (A1, group II), one patch per day, were applied to the shaved skin of rabbits and secured using USP adhesive tape (Johnson & Johnson limited, Mumbai). A 0.8% (v/v) aqueous solution of formaldehyde was applied as a standard irritant (group I). Its effect was compared with the test [19]. The animals were observed for any sign of erythema and edema for a period of 7 days and scored as reported by Draize et al. (1944) [20]. The Draize method of scoring was shown in Table 2.

**Table 2:** Draize evaluation of dermal reaction

**Scoring**	**Reaction**	
	**Erythema**	**Edema**
0	No erythema	No edema
1	Very slight erythema	Very slight edema
2	Well-defined erythema	Slight edema
3	Moderate to severe erythema	Moderate edema
4	Severe erythema	Severe edema

#### Anti-inflammatory study

The study was conducted in accordance with the Ethical Guide- lines for Investigations in Laboratory Animals and was approved by the Ethics Review Committee for Animal Experimentation of Osmania University. The anti-inflammatory activity and sustaining action of the drug-loaded drug in adhesive patches were evaluated using “carrageenan-induced hind paw edema” method developed by Winter et al. (1965) [21]. Wistar rats were used after being allowed to acclimatize for 1 week. Before the day of administration, rats were fasted overnight but were allowed access to water ad libitum. Eight rats weighing 180–220 g (6–8 weeks old) divided into two groups were used for the study. The backsides of rats were shaved 12 h before starting the experiments.

*Group – I* (Control group): Paw edema was induced by injecting 0.1 mL of a 1% w/v homogeneous suspension of carrageenan in double-distilled water [21] The volume of injected paw was measured immediately (0 h) and at 0.5, 0.75, 1, 2, 3, 4, 5, 6, 8, 10, 12, 16 and 24 h after injection using a IMCORP plethysmometer [22]. The amount of paw swelling with respect to initial volume was determined time to time. It is obtained by subtracting volume of injected paw at time ‘0’ from volume of injected paw at time ‘t’ divided by volume of injected paw at time ‘0’.

*Group – II (Test):* Treated similar to control group except that patches were applied half an hour before sub-plantar injection of carrageenan. Percent (%) inhibition of edema produced by each patch- treated group was calculated against the respective control group using the following formula

(1) $$\begin{array}{c} \%\;\mathrm{Inhibition}=\%\;\mathrm{edema}\left(\mathrm{control}\right)–\%\;\mathrm{edema}\left(\mathrm{drug}\right)\\ \;/\%\mathrm{edema}\left(\mathrm{control}\right)\times 100 \end{array}$$

## Results and discussion

### Evaluation of placebo silicone patches

The placebo patches using six grades of silicone polymer were prepared. The patches were smooth, flexible and uniform. The S_1_ patches were ruled out because during preparation of patches after drying the polymer completely lost its adhesive property.

An ideal adhesive polymer for drug-in-adhesive system is one that exhibit greater adhesion value. The initial screening of the silicone adhesives was done by ball tack test and peel strength of patches. The peel test, ball test and adhesive mass test values of all placebo silicone patches of different thicknesses were as shown in Table 3. The ball test and peel test values for different formulations of thickness 200 μm were in the following order: S3 > S6 > S5 > S2 > S4. Hence, S_6_ and S_3_ polymers showed better peel adhesion and ball test values hence, selected for further study.

**Table 3:** Adhesive mass values, ball test and peel test values for placebo silicone patches

**Parameter and thickness**	**Polymer type**				
	**S**_**2**_	**S**_**3**_	**S**_**4**_	**S**_**5**_	**S**_**6**_
***Ball tack test***^a^	4	8	0	5	8
***Peel test (Kg/cm*)**^b^	0.384	0.646	0.0938	0.495	0.645

(S_2_, S_3_, S_4_, S_5_ and S_6_ represents different Silicone polymers used; a = 3 and b = 5).

### Evaluation of drug loaded silicone patches

#### Solubility of drug in pure silicone adhesives

The solubility of Diclofenac Diethylamine in S_3_ and S_6_ was tested. The solutions that remained clear after 24 h were coated over the release liner. Results were shown in (Table 4). The Polymeric adhesive S_3_ only showed clear solution with 1% drug concentration. The results indicated low solubility of Diclofenac Diethylamine in Silicone adhesives and stresses on the need for the solubilizers for solubilization of drug.

**Table 4:** Solubilization summary of drug loaded silicone polymers

**Formulation code**	**Drug concentration**	**Solubilizer concentration**	**Observation**
**DS**_**3**_	5%	-	Clear solution was not formed.
**DS**_**3**_	3%	-	Clear solution was not formed.
**DS**_**3**_	2%	-	Clear solution was not formed.
**DS**_**3**_	1%	-	Clear solution indicating solubilization of drug
**DS**_**3**_	0.5%	-	Clear solution indicating solubilization of drug.
**DS**_**6**_	1%	-	Clear solution was not formed.
**DS**_**3**_**E**_**1**_	5%	5% PEG-400	Clear solution was not formed.
**DS**_**3**_**E**_**2**_	3%	5% PEG-400	Clear solution was not formed.
**DS**_**3**_**E**_**3**_	2%	3% PEG-400	Clear solution was formed.
**DS**_**6**_**E**_**4**_	1%	3% PEG-400	Clear solution was formed.
**DS**_**6**_**E**_**5**_	2%	3% PEG-400	Clear solution was not formed.
**DS**_**3**_**G**_**1**_	5%	5% PG	Clear solution was not formed.
**DS**_**3**_**G**_**2**_	2%	5% PG	Clear solution was formed.
**DS**_**3**_**G**_**3**_	1%	2% PG	Clear solution was formed.
**DS**_**3**_**O**_**1**_	3%	2% OLA	Clear solution was not formed
**DS**_**3**_**O**_**2**_	2%	2% OLA	Clear solution was not formed
**DS**_**3**_**O**_**3**_	1%	2% OLA	Clear solution was formed
**DS**_**3**_**I**_**1**_	4%	2% ISA	Clear solution was not formed
**DS**_**3**_**I**_**2**_	3%	2% ISA	Clear solution was not formed
**DS**_**3**_**I**_**3**_	1%	2% ISA	Clear solution was formed
**DS**_**3**_**M**_**1**_	1%	2% IPM	Clear solution was not formed
**DS**_**3**_**P**_**1**_	1%	2%Pharmasolve	Clear solution was not formed
**DS**_**3**_**O**_**4**_**I**_**4**_	3%	5% OLA & 5% ISA	Clear solution was formed.

DS: drug with silicone polymer; S_3_: Silicone polymer grade, S_3_; S_6_: Silicone polymer grade S_6_; OLA: oleic acid; ISA: Iso stearic acid; IPM: Isopalmitic acid; PEG 400: Polyethylene glycol −400; PG: Propylene glycol.

### Solubility enhancement techniques

#### Solubility of Drug in Silicone adhesives in the presence of solubilizers

Two solubilizers namely PEG - 400 and PG were tested to increase the solubility of Diclofenac Diethylamine in Silicone adhesives. Though PEG - 400 and PG increased Diclofenac Diethylamine solubility in water [9], their role to solubilize the drug in Silicone adhesive was abortive. Table 4 shows the drug concentration and solubilizer concentration used. Except few, all the solutions showed turbidity. In case of DS_3_E_3_ a clear solution was formed with 2% drug and 3% PEG - 400 while, in formulations containing S_6_ polymer (DS_6_E_5_) a clear solution was not formed with 2% drug and 3% PEG - 400. Similar to PEG - 400, PG showed slight improvement in solubility of drug in S_3_ polymer.

From solubility studies, it can be concluded that compared to S_6_, S_3_ polymer showed solubilization of DDEA to some extent. So, S_3_ polymer was chosen for further study.

#### Solubility of Drug in Silicone adhesives in the presence of oils

As formulations with PEG - 400 and PG showed little/no improvement in solubility, various oils namely oleic acid (OLA), IsoStearic acid (ISA), Pharmsolve® and Isopropyl Myristate (IPM) were tested for their ability to improve solubility of drug using the method described in experimental section. The formulations which remained clear after 24 h were coated over release liner. Among various oils tested, OLA and ISA were promising. However, only 1% drug was solubilized in both the cases (DS_3_O_3_ and DS_3_I_3_) While, IPM and Pharmsolve® did not even solubilize 1% drug. Combination of solubilizers was also tested but, only 3% drug solubilization was achieved in S_3_ polymeric adhesive at 10% solubilizer concentration (OLA and ISA, 5% each).

**Figure 1:**
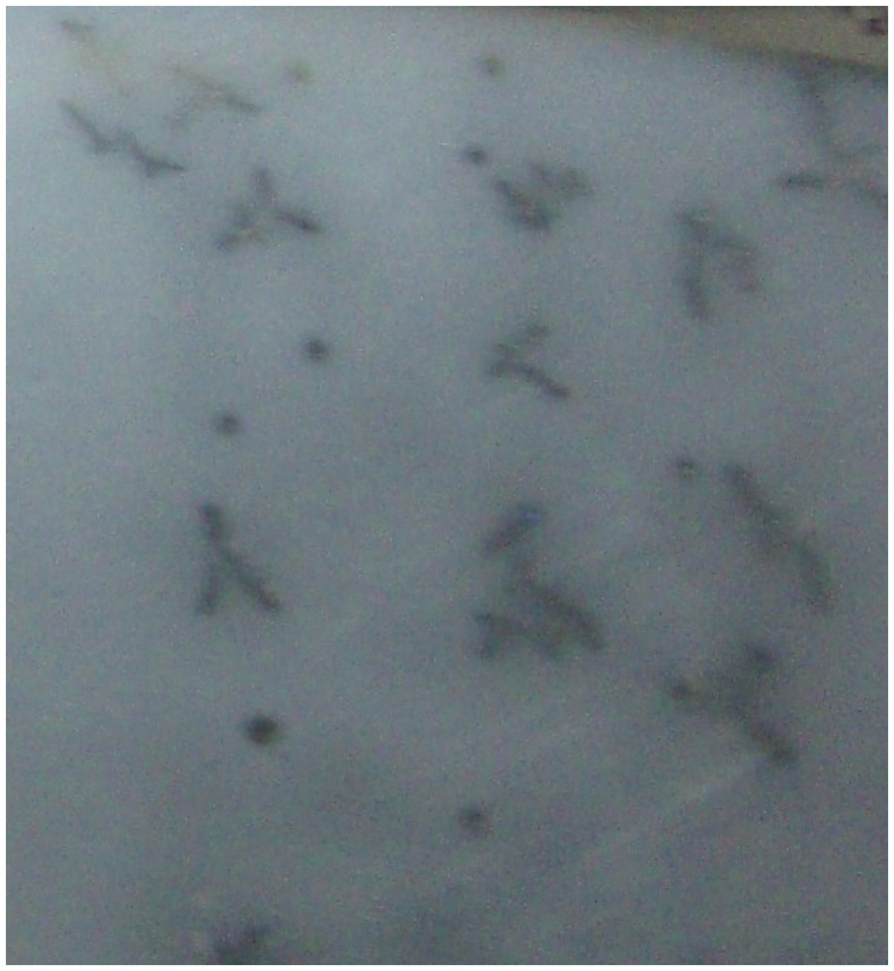
**Photograph of Silicone patch, DS**_**3**_**O**_**1,**_**showing crystal formation.**

**Figure 2:**
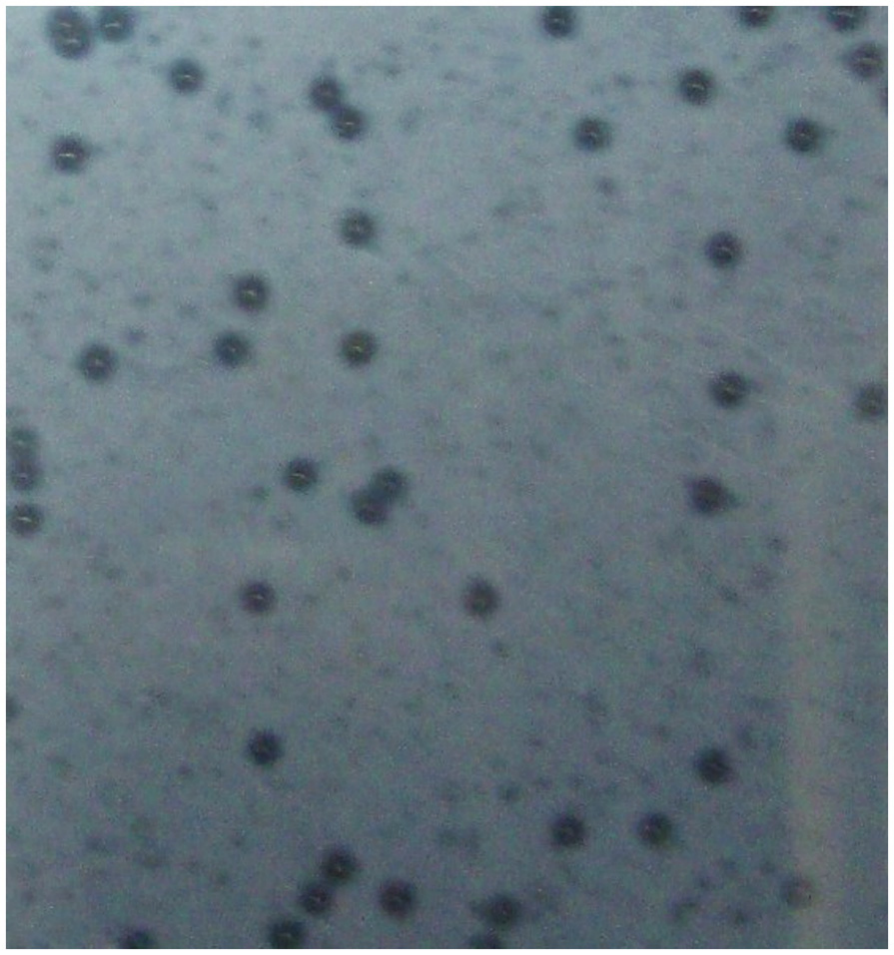
**Photograph of Silicone patch, DS**_**3**_**I**_**3,**_**showing crystal formation.**

The prepared patches (DS_3_ with 1% drug, DS_3_E_3_, DS_6_E_4_, DS_3_G_2_, DS_3_G_3_, DS_3_O_3_, DS_3_I_3_ and DS_3_O_4_I_4_) were uniform. However, after 10 days patches with additives OLA and ISA (DS_3_O_3_, DS_3_I_3_ and DS_3_O_4_I_4_) ended up with formation of crystal growth. Figure 1 and Figure 2 shows crystallization in patches DS_3_O_3_ and DS_3_I_3,_ respectively.

In case of formulations containing PEG - 400 and PG as additives, though the patches showed no crystallization, the solutions after 10 days took oil like consistency due to formation of oil globules in the solution resulting in loss of adhesion (Table 5).

**Table 5:** Crystallization summary of drug loaded silicone patches with or without solubilizers

**Formulation code**	**Drug concentration**	**Solubilizers concentration**	**Patch Observation after 10 days**	**Solution observation after 10 days**
**DS**_**3**_	0.5%	-	No sign of crystallization	Same as first day
**DS**_**3**_	1%	-	No sign of crystallization	Same as first day
**DS**_**3**_**E**_**3**_	2%	3% PEG-400	No sign of crystallization	Oil globules were formed and the solution turned oily
**DS**_**6**_**E**_**4**_	1%	3% PEG-400	No sign of crystallization	Oil globules were formed and the solution turned oily
**DS**_**3**_**G**_**2**_	2%	5% PG	No sign of crystallization.	Oil globules were formed and the solution turned oily
**DS**_**3**_**G**_**3**_	1%	2% PG	No sign of crystallization.	Oil globules were formed and the solution turned oily
**DS**_**3**_**O**_**3**_	1%	2% OLA	Crystallization was seen.	Same as first day
**DS**_**3**_**I**_**3**_	1%	2% ISA	Crystallization was seen.	Same as first day
**DS**_**3**_**O**_**4**_**I**_**4**_	3%	5% OLA & 5% ISA	Crystallization was seen.	Same as first day

The patches which did not contain any additives remained clear even after 10 days hence considered stable. Among various formulations prepared, DS_3_ containing 1% drug was chosen for further study.

### *Ex vivo* skin permeation study

The DS_3_ patch containing 1% drug was chosen for conducting permeation study. The cumulative amount of drug permeated (CPD) at the end of 24 h was found to be 19.573 mcg/cm^2^ (Table 6). Though the amount of drug permeated was low, the percentage cumulative amount of drug permeated was 23.209%. The low CPD value might be due to less amount of drug (1%) in the patch.

**Table 6:** **Permeation study of formulation DS**_**3 **_**and DA**

**S. No**	**TIME (h)**	**DS**_**3**_		**DA**	
		**CDP (μg/cm**^**2**^**)**	**% CDP**	**CDP (μg/cm**^**2**^**)**	**% CDP**
1.	0	0	0	0	0
2.	2	1.185	1.405138	5.934	0.84051
3.	4	2.6843	3.182964	12.847	1.819688
4.	6	4.2813	5.07664	19.131	2.709773
5.	8	5.9842	7.095889	25.819	3.657082
6.	10	7.7583	9.199565	31.824	4.507649
7.	12	9.3142	11.04451	38.119	5.399292
8.	14	11.042	13.09328	45.248	6.409065
9.	24	19.573	23.20909	75.692	10.72125

CDP: Cumulative drug permeated; % CDP: percent cumulative drug permeated; DA: drug with Acrylic polymer alone.

### Evaluation of drug loaded acrylic patches

The extensive solubilization study conducted revealed that the silicone polymer is unsuitable for achieving very high concentrations of DDEA. Hence, solubility of drug in acrylic adhesive was tested by method described under experimental section I of IIIA. It was noticed that drug concentrations up to 20% was solubilized without use of any additives. The prepared patches were also stable after 10 days and did not showed crystal formation. While 25% of drug polymeric solution resulted in turbidity (Table 7). This might be because of drug loading greater than the saturation solubility of the drug in the adhesive used. However, the concentration of drug was fixed at 10% for further study since the formulation being studied is intended for topical use. The Ball test and peel strength values for DA were shown in Table 7.

**Table 7:** Solubilization summary, peel test, ball test and adhesive mass value for acrylic adhesive patches

**Polymer**	**Drug concentration**	**Observation**	
A	10%	Clear solution formed	
A	15%	Clear solution formed	
A	20%	Clear solution formed	
A	25%	Clear solution was not formed	
**Parameter evaluated**		**DA**	
Peel test (Kg/cm)		0.9306	
Ball test		8	

(n is equal to 5 and 3 for Peel test and Ball test respectively).

### *Ex vivo* skin permeation study

Skin permeation of Diclofenac Diethylamine was studied using DA patch containing 10% drug. Study was conducted for 24 h without using permeation enhancer. The cumulative amount of drug permeated into the receptor compartment was 75.692 mcg/cm^2^ after 24 h (Table 6) that represents 10.72% of the total drug placed in the donor compartment.

Though the CDP of DA was significantly greater than CDP of DS_3_ the percent drug permeated was high in case of DS_3_ (23.209%). The CDP of DA was found to be significantly high because the drug concentration in DA was 10 times greater than that of DS_3_.

### Evaluation of drug loaded combinational patches

Studies on Silicone adhesives revealed poor solubilization capacity and high percent cumulative drug permeation (%CDP) value whereas; acrylic polymers solubilized higher concentrations of drug but exhibited less%CDP. Hence an attempt was made to combine high%CDP property of Silicone and greater drug solubilization property of acrylic polymer by fabricating a drug formulation with combination of adhesives.

The placebo solutions containing different proportions of Silicone and Acrylic adhesives were prepared and used as reference for checking the solubility of drug in combination of adhesives. The combinations from C_1_ to C_5_ showed similar consistency as compared to respective placebo patches after addition of drug. Table 8 shows the Solubility data of different combinations of Silicone and acrylic with 10% drug. The results indicated that minimum 50% acrylic polymer is required in the formulation to achieve 10% drug solubility (As acrylic polymer alone can solubilize 20% drug without any solubilizer as mentioned earlier, it is evident that 50% acrylic polymer is sufficient to solubilize 10% drug). Hence, formulations C_1_, C_2_, C_3_, C_4_ and C_5_ were used for further study.

**Table 8:** Solubility of drug in combinational patches

**Formulation code**	**Ratio of Silicone: Acrylic**	**Targeted drug concentration**	**Solubility observation**
C_1_	10: 90	10%	YES
C_2_	20: 80	10%	YES
C_3_	30: 70	10%	YES
C_4_	40: 60	10%	YES
C_5_	50: 50	10%	YES
C_6_	60: 40	10%	NO
C_7_	70: 30	10%	NO
C_8_	80: 20	10%	NO
C_9_	90: 10	10%	NO

C: combination patches with Acrylic and Silicone adhesives.

### Ex vivo skin permeation experiment

**Figure 3:**
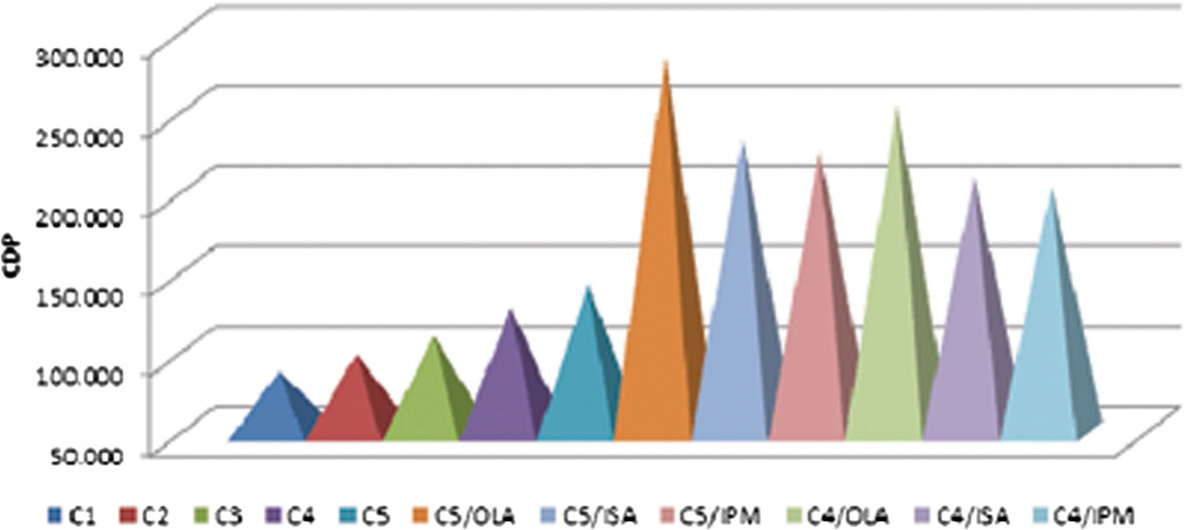
**Graph showing cumulative amount of drug permeated (CDP) at the end of 24 h with and without permeation enhancers for combinational patches (C**_**4**_**/OLA, C**_**4**_**/ISA and C**_**4**_**/IPM reprsents C**_**4**_**patch with oleic acid (OLA), Isostearic acid and Isopalmitic Myristate as permeation enhancers, respectively.** Similar in case of C_5_ combinational patches).

In all the combinational patches, the CDP of combinational patches was higher than that DA patches which contains Acrylic polymer alone. Figure 3 shows the amount of drug permeated at the end of 24 h with and without combinational patches. The CDP of different combinational patches were in the following order:

$$C_{5}>C_{4}>C_{3}>C_{2}>C_{1}\text{.}$$

The above order once again reflected the previous results i.e. with increase in amount of Silicone polymer the amount of CDP increased. Among all five formulations C_5_ displayed high CDP value due to its high Silicone content (50% of the total polymer).

**Figure 4:**
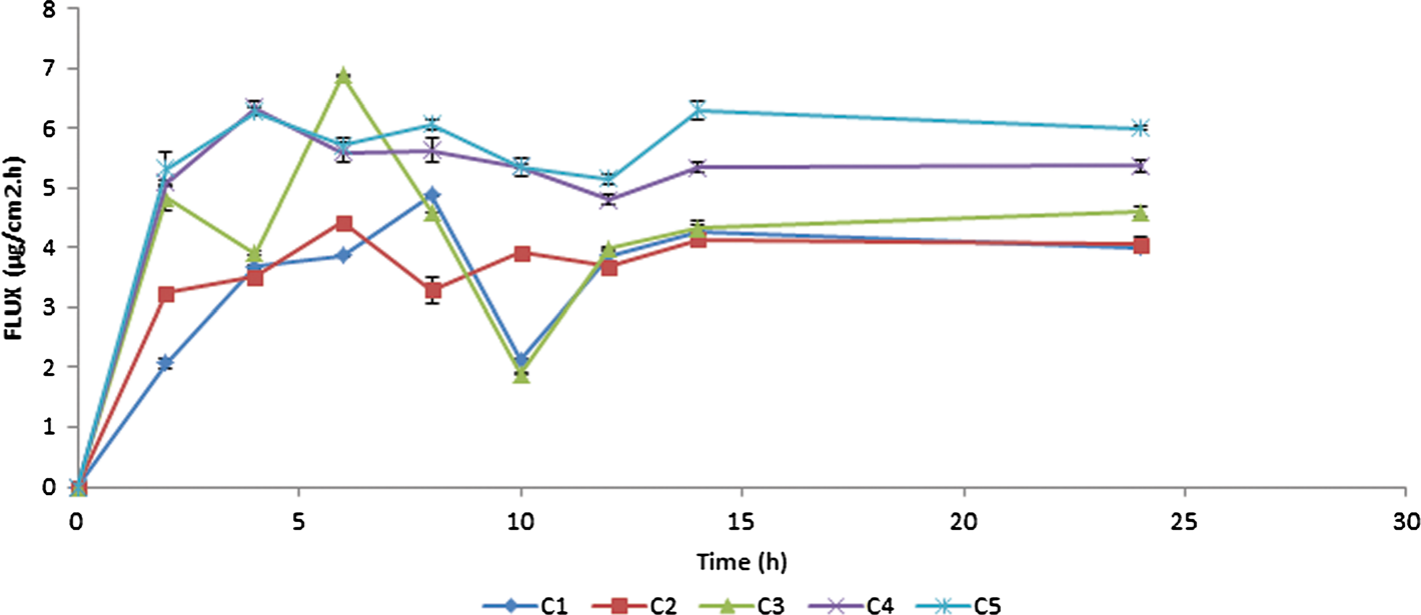
**Graph showing the plot between Flux (μg/cm**^**2**^**.h) and time (h) for C**_**1**_**, C**_**2**_**, C**_**3**_**, C**_**4**_**and C**_**5**_**patches (data represented as mean ± S.D).**

Figure 4 shows plot between flux and time for all the five combinations. The graph showed little/less variation in the flux between different time intervals for C_4_ and C_5._ Moreover, the CDP was found to be relatively high for these two formulations. Hence, the formulations C_4_ and C_5_ were chosen for further study.

### Effect of permeation enhancers on the permeation of DDEA

Three permeation enhancers namely, OLA, ISA and IPM were used at 5% concentration. The cumulative amount of drug permeated at the end of 24 h was represented in Figure 3. The permeation data revealed greater penetration enhancing capability of OLA than ISA and IPM. This is in line with the result reported where OLA increased the permeation of DDEA by 7–9 folds (Hussain Shah et al. 2012) [10]. Thus, it can be concluded that vehicles used here were predominantly influencing the partition of the drug into the skin. Hence, C_4_/OLA and C_5_/OLA which exhibited greater CDP among all were chosen as optimized formulations.

**Figure 5:**
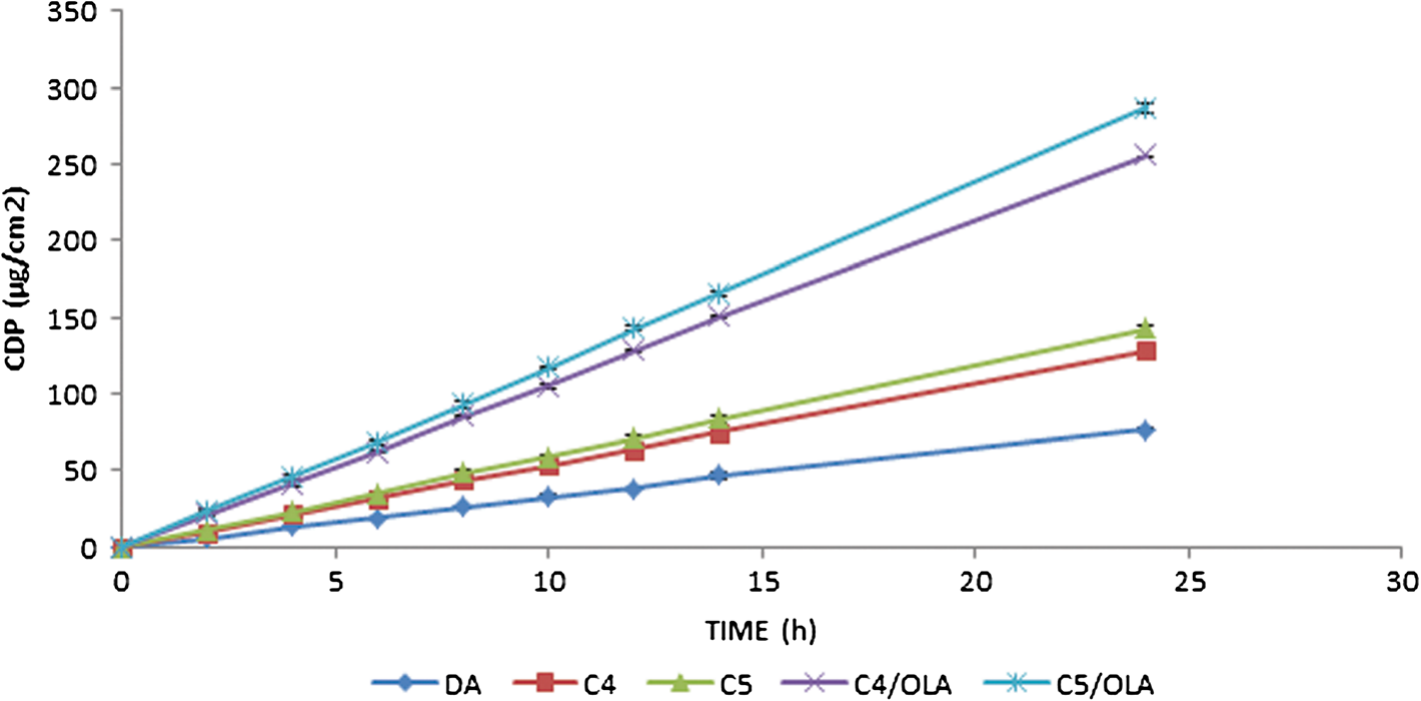
**Graph showing the plot between cumulative amount of drug permeated (CDP, μg/cm**^**2**^**) and time (h) for DA, C**_**4,**_**C**_**5**_**, C**_**5**_**/OLA and C**_**4**_**/OLA (data represented as mean ± S.D).**

Figure 5 shows the plot between CDP and time for different formulations. From the graph, it can be predicted that C_4_ and C_5_ showed high CDP compared to DA indicating more drug permeation capacity compared to individual Acrylic formulations. OLA application as a permeation enhancer was well justified as significant increase in CDP value was observed compared to patches without enhancers.

### Dissolution study of patches

**Figure 6:**
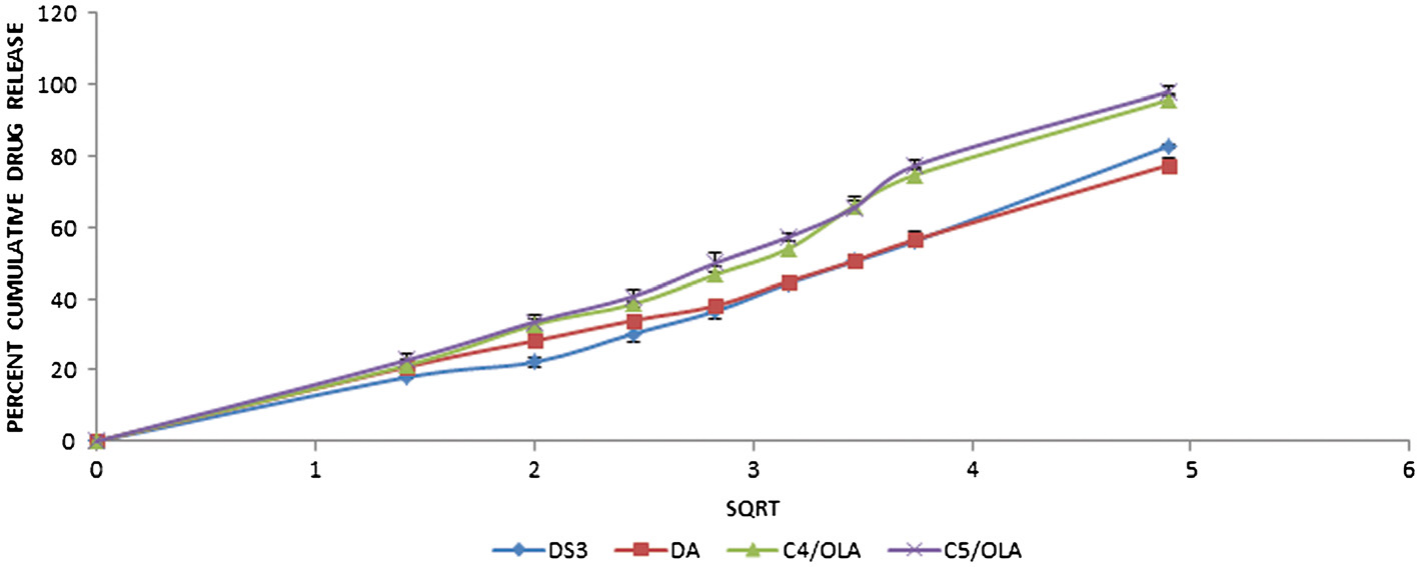
**Graph showing the Higuchi plot for DS**_**3**_**, DA, C**_**5**_**/OLA and C**_**4**_**/OLA (data represented as mean ± S.D).**

*In vitro* release profile is an important tool that predicts in advance how the drug will behave in vivo. Thus, we can eliminate the risk of hazards during experimentation in living system. Five patches, DS_3_, DA, C_4_/OLA and C_5_/OLA were studied for drug release. The study was conducted for a period of 24 h. The percent drug release (Figure 6) was found to be in the following order:

$$C_{5}/\mathrm{OLA}>C_{4}/\mathrm{OLA}>{\mathrm{DS}}_{3}>\mathrm{DA}$$

The dissolution values revealed that formulations containing OLA exhibited greater percent cumulative drug release (%CDR) than DS_3_ and DA. This might be due to increased solubility of poorly soluble drug, DDEA, in water due to OLA. Among C_5_/OLA and C_4_/OLA the formulation containing greater portion of silicone polymer, C_5_/OLA, showed greater%CDR.

### Release kinetics

The dissolution data of C_4_/OLA and C_5_/OLA was put forth for release kinetic studies. Based on high R^2^ value it was shown that drug release from the formulations followed Higuchi pattern of drug release, with R^2^ value 0.978 for C_4_/OLA and 0.981 for C_5_/OLA, (Figure 6) where drug diffusion through the polymeric system was the main mechanism. The ‘n’ value from the korsemeyer-peppas plot revealed non-fickian/anomalous diffusion pattern (n>0.5).

### Stability study

**Figure 7:**
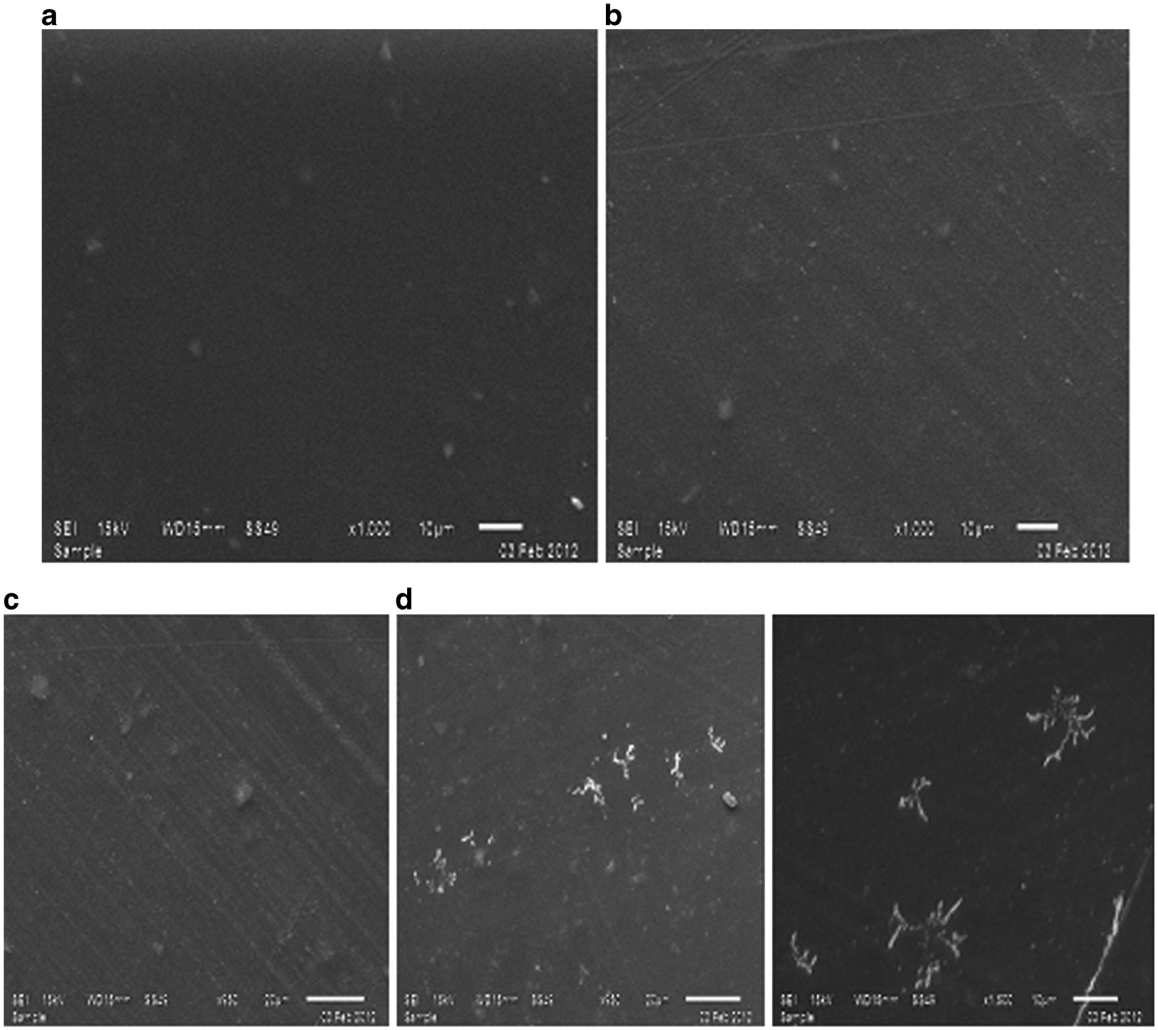
**SEM micrographs of optimized formulations before and after stability study: (a) and (c) represent C**_**4**_**/OLA and C**_**5**_**/OLA patches before stability, respectively; (b) and (d) represent C**_**4**_**/OLA and C**_**5**_**/OLA patches after stability, respectively.**

**Figure 8:**
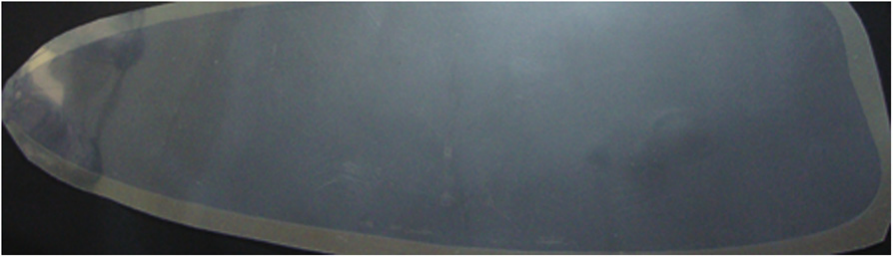
**Photograph of the patches C**_**4**_**/OLA after stability.**

**Figure 9:**
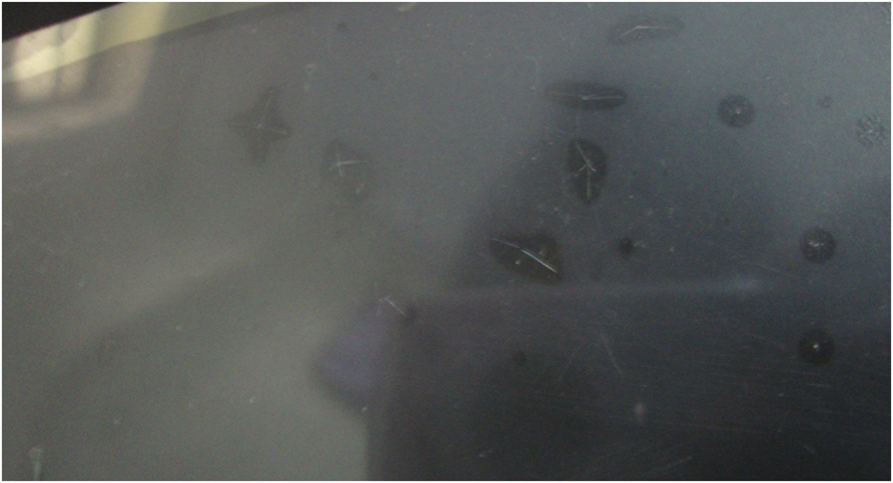
**Photograph of the patches C**_**5**_**/OLA after stability.**

The formulations C_5_/OLA and C_4_/OLA were kept for 3 month stability study. During stability study in case of C_5_/OLA, crystallization (Figure 7) was observed which might be due to saturation of drug solubility which resulted in slow precipitation of drug. This is also reflected in its drug content shown in Table 9 where the percent drug content of the formulation kept on decreasing. Such a saturated matrix is unstable and the drug will recrystallize in such systems over time [23-25]^.^ Recrystallization may however not be apparent immediately after manufacture because of the relatively low diffusion coefficients of drug in such highly viscous systems and the requirement of nucleation for the initiation of crystallization. Figure 8 shows the photograph of the C_4_/OLA after stability with no crystals and Figure 9 shows photograph of the C_5_/OLA after stability with crystal formation.

**Table 9:** **Stability data for formulation C**_**4**_**/OLA and C**_**5**_**/OLA**

**Tested parameters**	**0 days**	**45 days**	**90 days**
**C**_**4**_**/OLA**			
Peel Strength (Kg/cm)	0.663	0.646	0.659
Drug content (%)	104.6	103.6	104.1
**C**_**5**_**/OLA**			
Peel Strength (Kg/cm)	0.654	0.649	0.652
Drug content (%)	101.64	98.86	94.38

(C_4_/OLA: C_4_ combination patch with oleic acid, OLA, as permeation enhancer; C_5_/OLA: C_5_combination patch with oleic acid, OLA, as permeation enhancer; n=3 for Drug content and n=5 for Peel strength).

The peel test of both the formulations showed no significant change during stability study indicating the sustainability of adhesive property of the polymeric combination.

However, in case of C_4_/OLA crystallization was not found and moreover the drug content remained stable representing robustness of the formulation during 3 month stability. Hence, the formulation C_4_/OLA was found to be the optimized formulation.

### Physical evaluation of optimized patches

**Figure 10:**
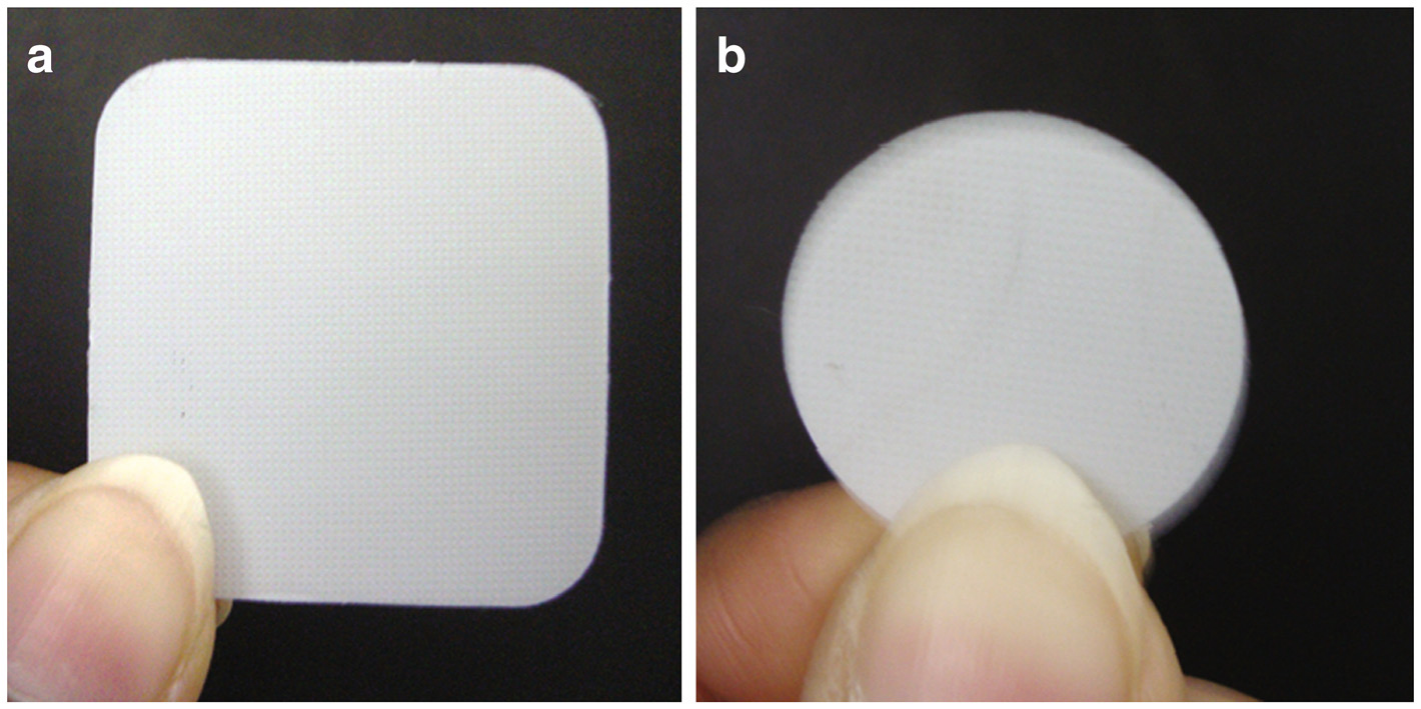
**Photograph of optimized C**_**4**_**/OLA patches both 10 cm**^**2**^**(a) and 3 cm**^**2**^**(b) patches.**

Figure 10(a) and 10(b) shows the original patch C_4_/OLA of sizes 10 cm^2^ and 3 cm^2^, respectively. The optimized formulation C_4_/OLA was tested for various physical parameters. The thickness (n = 5) of C_4_/OLA patches was found to be 181.63 ± 0.03 μm. Good weight uniformity among the batches was observed for all formulations and ranged from 214.33 – 216.35 mg. The results indicate that the process which was employed to prepare patches in this study was capable to produce patches with uniform drug content and minimal patch variability.

### Acute skin irritancy study

**Figure 11:**
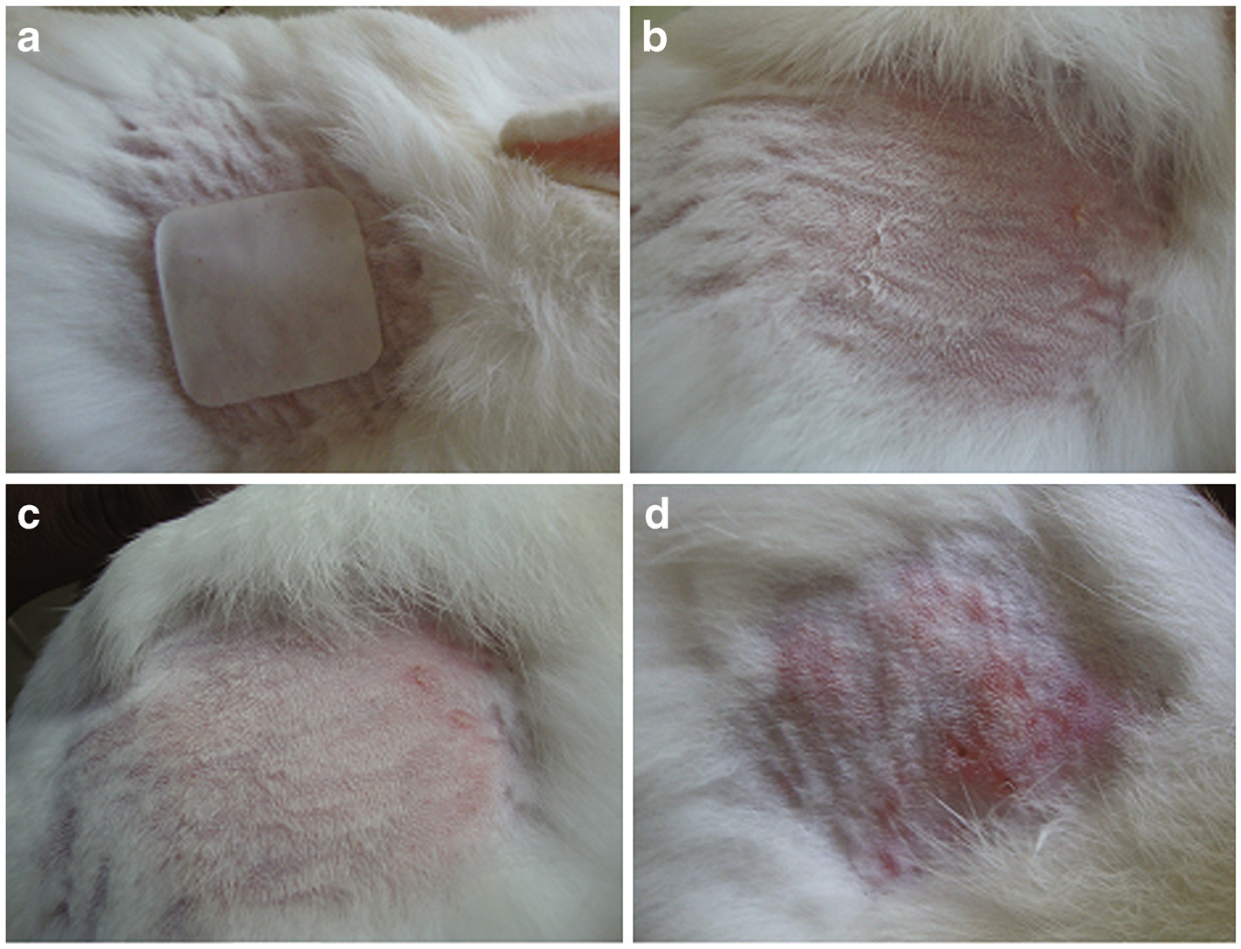
Images of skin irritancy study: (a) patch application to shaved area; (b) skin of rabbit at 0 day; (c) skin of test group rabbit after 7 days of patch application; (d) skin of standard group rabbit after 7 days.

The 7 day skin irritancy study revealed that the test formulation showed a skin irritation score (erythema and edema) of less than 1 (Table 10 & Figure 11). From the Draize method of scoring, the control animals showed severe erythema and moderate to slight edema whereas the test animals showed only very slight erythema and no edema on the site of application. According to Draize et al. (1944) [20] compounds producing scores of 2 or less are considered non-irritant [14]. Hence from the study, we can conclude that formulations are non-irritable to skin and safer for therapeutic use.

**Table 10:** **Acute skin irritancy data for C**_**5**_**/OLA (n = 4)**

**Day**	**Parameter**	**Standard**				**Test**			
		**1**	**2**	**3**	**4**	**1**	**2**	**3**	**4**
**Day 0**	**Erythema**	0	0	0	0	0	0	0	0
	**Edema**	0	0	0	0	0	0	0	0
**Day 7**	**Erythema**	4	4	3	4	0	1	1	1
	**Edema**	3	2	2	3	0	0	0	0

**(**C_4_/OLA: C_4_combination patch with oleic acid, OLA, as permeation enhancer).

### In vivo anti-inflammatory studies

The result of carrageenan induced paw edema test was shown in the Table 11. The table shows the data for the percent increase in edema with respect to initial volume and percentage inhibition of edema with respect to control during 24 h study for the test formulation. As shown in the table in case of control group animals, the mean percent increase in edema with respect to initial volume (Group –I) was 114.3 ± 15.0 at the end of 24 h which is because of swelling nature of carrageenan. While in case of test group animals the value is 0.37 ± 0.54 at the end of 24 h indicating that test patches, C_4_/OLA, are effective in inhibiting carrageenan induced inflammation. Moreover, the test group animals showed 99.68% inhibition of edema with respect to control after 24 h indicating the efficacy of the formulation during the period. The initial percent increase in edema with respect to initial volume in case of test group half an hour after the carrageenan induction was 0.4 ± 0.54 as opposed to Control group (3.57 ±1.08) indicating that the test patch, C_4_/OLA showed action from the first hour without any appreciable lag time. Throughout the study the percent increase in edema value with respect to initial volume for test group remained well below than the control group indicating the sustaining effect of the drug against carrageenan challenge.

**Table 11:** Paw edema data obtained on carrageenan induced rats half an hour after the patch application (data represented as mean ± S.D, n = 4)

**Time (h)**														
	**0.5**	**0.75**	**1**	**2**	**3**	**4**	**5**	**6**	**7**	**8**	**10**	**12**	**16**	**24**
**% Edema with respect to initial volume**														
**Control**	3.57 ±1.08	14.28 ± 2.05	35.71 ± 7.06	42.8 ±9.64	50.0 ± 12.95	60.7 ± 20.82	71.5 ± 19.72	83.0 ± 21.85	92.8 ± 11.05	100.0 ± 15.1	111.1 ± 18.6	121.4 ± 11.76	121.4 ±15.9	114.3 ± 15.0
**Test (C**_**4**_**/OLA)**	0.4 ± 0.54	5.6 ± 2.91	11.3 ± 5.83	18.5 ± 0.97	39.4 ± 4.16	54.1 ± 0.31	62.6 ± 1.44	52.7 ± 9.29	42.6 ± 0.39	36.2 ± 4.39	21.2 ± 2.68	15.6 ± 0.82	2.5 ± 2.49	0.37 ± 0.54
**% inhibition of Edema with respect to control**														
**Test (C**_**4**_**/OLA)**	88.80	60.78	68.36	56.78	21.20	10.87	12.45	36.51	54.09	63.80	80.92	87.15	97.94	99.68

## Conclusion

The extensive solubilization study conducted on Silicone adhesive polymers revealed their unsuitability in fabrication of DDEA transdermal patches alone as not more than 1% drug was solubilized even with high concentration of solubilizer. On the other hand, Acrylic polymer showed high drug loading and greater control releasing capacity hence, alone can be used for fabricating transdermal patches of DDEA. However, use of Acrylic alone requires greater amount of drug incorporation due to its low value of percent cumulative drug permitted (10.72%). Hence, the combinations of adhesives were tested with the objective combining the greater permeation capacity of Silicone polymer and greater drug loading capacity of Acrylic polymer. The combinational patches incorporating the both the desired properties were successfully prepared. Among various permeation enhancers tested OLA proved to be a good permeation enhancer as compared to ISA and IPM for DDEA. C_4_/OLA was found to be optimized formulation displaying robustness in stability. The skin irritancy study revealed the non-irritant nature of the C_4_/OLA patches and sustaining action of the patches were confirmed by anti-inflammatory test by carrageenan induced paw edema model. Thus, it can be concluded that an ideal of combination of adhesives would serve as the best choice, for fabrication of DDEA patches, for sustained effect of DDEA with better enhancement in permeation characteristics and robustness.

## Competing interests

The manuscript has no conflict of interest and there are no financial sources for many organizations and the work is purely part of student thesis work.

## Authors’ contributions

The author DPM helped in conceptual design of entire work, the author SP was responsible for the entire practical work, TP contributed to the interpretation of data obtained at various steps, the author BD helped in carrying out the studies involving animals and AKA helped in the calculation part and in preparation and follow up of the manuscript. All authors read and approved the final manuscript.
